# The Association of Certificated Blind Masseurs

**Published:** 1920-11-13

**Authors:** C. Mansell Moullin, Alfred Pearce Gould, Arthur Pearson

**Affiliations:** Col. R.A.M.C.T. 224-6-8 Great Portland Street, London, W. 1; 224-6-8 Great Portland Street, London, W. 1; President, National Institute for the Blind, and Association of Certificated Blind Masseurs. 224-6-8 Great Portland Street, London, W. 1


The Editor's Letter Box—(*continued*).

### The Association of Certificated Blind Masseurs.

*To the Editor of* The Hospital.

Sir,—The Association numbers 126 members—105 men and twenty-one women—all of whom are practically blind. Seventy-six of the men were in H.M.'s Services and lost their eyesight through injury or disease incurred in the war. The training they have received at St. Dunstan's and at the School of Massage attached to the National Institute for the Blind is of the most thorough description. It lasts for eighteen months or two years, and, after it is over, they have to pass the massage examination of the Chartered Society of Massage and Medical Gymnastics, under exactly the same conditions as sighted candidates. (At a recent examination a blind candidate from St. Dunstan's was returned at the head of the list, with distinction.) They have also to pass a special examination in remedial exercises, and (after another three months' training) in modern methods of treatment by Faradism, ionisation, radiant heat, etc., using a form of galvanometer specially adapted for the blind.

It is hardly necessary for us to point out that massage, as distinguished from mere rubbing, with which it is often confused much to its discredit, is a scientific method of treatment which can only be carried out efficiently by those who have had a thorough training. May we ask the members of the profession, when they recommend this method of treatment for their patients, to employ, whenever they can, those who have deserved so well of their country, and who, in spite of their handicap, are making such a splendid effort to make good?

We need hardly say that no member of the Association is ever allowed to treat patients without the consent and advice of a registered medical practitioner.

Forty-nine of the members of the Association are living in London or its immediate neighbourhood. The rest (with the exception of a few who have gone to the Colonies) are in the provinces. A list, with names and addresses, will be sent with pleasure by the Secretary, Mrs. F. Chaplin Hall, on receipt of a postcard.—We are, Sir, yours faithfully,

C. Mansell Moullin, C.B.E., F.R.C.S., Col. R.A.M.C.T.

Alfred Pearce Gould, K.C.V.O., C.B.E., M.S., F.R.C.S.

Arthur Pearson, President, National Institute for the Blind, and Association of Certificated Blind Masseurs.

224-6-8 Great Portland Street, London, W. 1,
November 6, 1920.

